# Optimization of biogas production from anaerobic co-digestion of fish waste and water hyacinth

**DOI:** 10.1186/s13068-023-02360-w

**Published:** 2023-07-06

**Authors:** Hortence Ingabire, Milton M. M’arimi, Kirimi H. Kiriamiti, Boniface Ntambara

**Affiliations:** 1 Department of Mechanical, Production and Energy Engineering, School of Engineering, Moi University, P. O Box 3900-30100, Eldoret, Uasin Gishu County Kenya; 2 Africa Centre of Excellence II in Phytochemicals, Textiles and Renewable Energy (ACE II PTRE), Moi University, P. O Box 3900-30100, Eldoret, Uasin Gishu County Kenya; 3 Department of Chemical & Process Engineering, School of Engineering, Moi University, P. O Box 3900-30100, Eldoret, Uasin Gishu County Kenya

**Keywords:** Biogas production, Anaerobic co-production, Biogas optimization, Biodegradable fish waste, Water hyacinth digestion

## Abstract

Many fresh water bodies face a great challenge of an invasive weed called water hyacinth (WH) which has great impacts on the environment, ecology, and society. Food and Agriculture Organization (FAO) estimates that over nine million tons of Fish wastes (FW) are thrown away each year. The fish waste generated poses environmental and health hazards because in most cases it is either disposed into pits or discarded onto the open grounds. Both WH and FW are potential substrates for biogas production. However, utilization of FW substrate alone has a limitation of producing a lot of amounts of volatile fatty acids (VFAs) and ammonia. Their accumulation in the digester inhibits substrate digestion. Consequently, as stand-alone it is not suitable for anaerobic digestion (AD). This can be overcome by co-digestion with a substrate like WH which has high carbon to nitrogen (C/N) ratio prior to biodigestion. Experimental variable levels for biogas were substrate ratio (WH:FW, 25–75 g), inoculum concentration (IC, 5–15 g/250 mL), and dilution (85–95 mL). Design-Expert 13 was used for optimization and results analysis. Response surface methodology (RSM) was used to examine the effects of operating parameters and identify optimum values for biogas yield. Optimum values for maximum biogas with the highest methane yield of 68% were found to be WH:FW ratio, 25:75 g, 15 g of IC, and 95 mL for dilution. The yield was 16% and 32% greater than FW and WH mono-digestion, respectively. The biogas yield was expressed as a function of operating variables using a quadratic equation. The model was significant (*P* < 0.05). All factors had significant linear and quadratic effects on biogas while only the interaction effects of the two factors were significant. The coefficient of determination (*R*^2^) of 99.9% confirmed the good fit of the model with experimental variables.

## Background

Biogas is produced from various organic wastes and used as energy source worldwide. It aids in attaining sustainability by providing access to modern, clean energy that is inexpensive, and dependable and fights climate change and its effects by limiting emissions [1–3]. According to FAO, approximately 9.1 million tons of fish waste are thrown annually. Consequently, fish by-products are now a global problem to the long-term viability of fish aquaculture [4]. WH is one of the most invasive water weeds in the world that thrives in freshwater bodies and has spread to most nations, has detrimental impacts on the environment, the ecology, and society [5–9]. It creates mats that obstruct waterways, makes fishing impossible, limits water flow, degrades water quality by obstructing sunlight from penetrating the water and lowering oxygen levels in the water, wipes out aquatic life like fish, and significantly reduces biodiversity. The waste generated is either disposed into pits or discarded onto the open ground which result in environmental pollution and health hazards [10–14]. AD of FW and WH can be used to enhance biogas generation. WH has high cellulose, low lignin contents, and high C/N ratio while FW is rich in lipids, proteins and contains easily biodegradable organic matter [2, 5, 12]. When digested alone, FW produce a lot of ammonia and volatile fatty acids (VFAs). Their accumulation in the digester inhibits substrate digestion. This makes FW not a suitable substrate for biogas production through anaerobic digestion (AD). A plausible way to overcome the limitation is through co-digestion with WH which has a high C/N ratio. Furthermore, optimization of the operating variables is necessary to overcome the biogas inhibition of FW [5, 6]. The application of co-digestion to balance the C/N ratio, improve gas and methane generation has been reported by previous studies [7]. The process enhances good synergy which encourages bacteria activity [11]. In addition to promoting co-digestion, the biogas yield can be improved by optimizing the process variables such as organic loading rate (OLR), inoculum concentration (IC), pH, dilution, carbon to nitrogen (C/N) ratio, substrate ratio, retention time, dilution, and temperature [6]. Nalinga and Legonda [15] demonstrated that anaerobic co-digestion of FW and WH feedstock increased the materials' digestibility and biogas yield. Tasnim et al. [16] conducted a study on anaerobic co-digestion of kitchen waste, cow manure, and WH. Their research revealed that kitchen waste combined with WH and cow manure was a source of biogas energy for both residential and commercial energy needs. Katima [8] studied biogas generation from WH by investigating the impact of substrate concentration (5 to 30 g/L), particle size (1–3 mm), and incubation period (1–6 days). The highest methane (72.53%) was generated within 5 days of incubation at a substrate concentration of 25 g/L and particle size less than 1 mm of WH. Usman [17] conducted a test on optimum biogas production from sugar cane and rice husk with the cellulolytic fungus by varying factors such as water, fungus concentration, and temperature. The optimum biogas of 500 cm^3^ was produced at the optimal values of 25 cm^3^ of water, 0.6 g of fungus, and a temperature of 33 ℃. Chanathaworn [18] has researched optimization conditions for biogas production from WH and earthworm bedding wastewater by varying particle size (0.3–1.5 cm), TS (4–12%), and pH. Optimum biogas of 35.50% was obtained at 8% of TS, 0.3 cm particle size, and 7.0 initial pH. Sandhu and Kaushal [19] applied the response surface technique to optimize the variables of co-digestion such as temperature, pH and concentration of wastes. It was also observed that the rate of biogas yield is greatly affected by many factors such as temperature and total solid concentration. There is no documentation in literature for the optimization of the anaerobic co-digestion of FW and WH. Some of the applications for biogas include; lighting, cooking, heating, etc. [19, 20]. The FW and WH are produced in large volumes in many countries. They are affordable, available, sustainable, and renewable; consequently, the successful utilization of WH and FW for biogas generation can have a significant impact. The main goal of this research study was to determine the optimal conditions for optimizing the production of biogas in AD by evaluating the impacts of inoculum concentration (IC), substrate (WH:FW) ratio, and dilution (water content) on biogas production by design of Expert (DOE) using RSM approach.

## Materials and methods

### Substrate collection and preparation

**Fig. 1:**
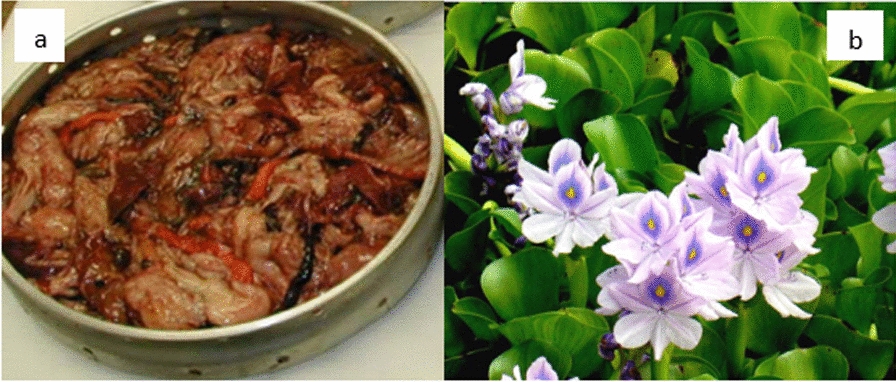
Illustration of fish waste (**a**) and water hyacinth (**b**) as feedstocks for biogas production

The water hyacinth used was sourced from Lake Victoria in Kisumu County. It was washed to remove unwanted impurities, cut into small pieces, and mashed using laboratory mortar to increase its biodegradability for microbial activity. Thereafter, they were put in a plastic collector and stored in a refrigerator for further use. The fish wastes (mostly fish intestines) used in this experiment were collected from the fish point, Eldoret, Kenya, and chopped into small pieces. The inoculum used in the experiment was freshly digested cow dung which was collected from the Moi University biogas plant, in Eldoret, Kenya as shown in Fig. 1. Fresh bio-digested cow dung was used as an inoculum because it contains active bacteria.

### Analytical methods

The FW, WH, and inoculum were analyzed for Moisture content (MC), Total solids (TS), Volatile solid (VS), Ash content, pH, and carbon to nitrogen (C/N) ratio using standard methods [12–14, 18, 21]. The characteristics of the feedstock are presented in Table 1.

**Table 1:** Characteristics of the feedstock

Characteristics	FW	WH	Inoculum
MC (%)	61.78	94.4	89.67
TS (%)	38.21	5.6	10.33
VS (%)	93.94	83.3	74.9
Ash content (%)	0.52	16.7	25.1
Total carbon (%)	54.2	42.7	35.91
Total nitrogen (%)	9.2	2.0	1.53
C/N ratio	5.89	21.35	23.47
pH	6.5	7.2	6.8

### Operating procedure

**Fig. 2:**
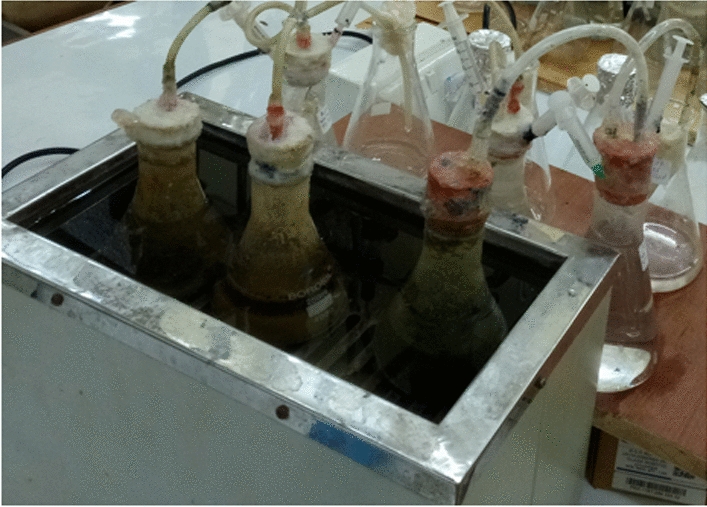
Illustration of the experimental setup

Conical flasks of 250 mL were used for batch digestion tests of biogas generation. According to the experimental plan, the substrates were fed into the reactor at varied ratios of IC (1.6–18.4 g/250 mL), substrate ratio (8–92 g), and dilution (81.6–98.4 mL). The biogas production set up is shown in Fig. 2.

**Fig. 3:**
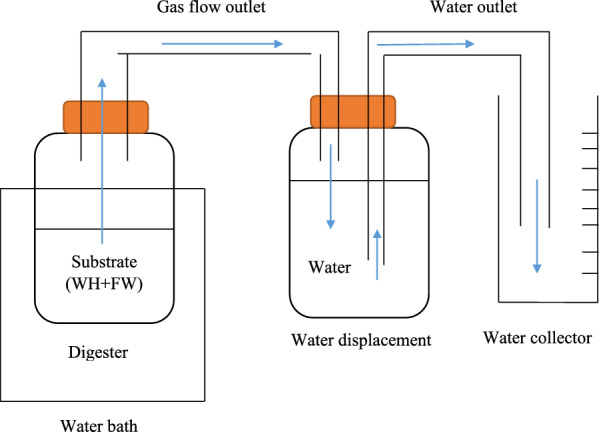
Overview of biogas production setup

The co-digestion was quantified by substrate ratio (based on 100 g). Biogas production was measured by water displacement method as illustrated in Fig. 3. The entire investigation was conducted at mesophilic temperature (37 °C). A gas detector was used to measure the methane content from the gas sampling bags.

### Experimental design and optimization

Design Expert 13 software which contains Central Composite Design (CCD), Analysis of Variance (ANOVA), and Response surface Methodology (RSM) was used for optimization. The CCD was used to determine the level of variable inputs and establish the optimum number of experimental runs, ANOVA was used for the analysis of the regression coefficient and the prediction equation, and to show how the variables interacted and RSM was used to examine the relationship or interaction between variables and the response and to estimate the optimum surface area of optimal values of the response. The polynomial equation was illustrated in 2D (two-dimensional) contour plots and 3D (three-dimensional) using response surface plots. Three factors: (*X*_1_: Substrate ratio (WH:FW, 25–75 g), *X*_2_: Inoculum concentration (IC, 5–15 g/250 mL), and *X*_3_: Dilution (85–95 mL)) were investigated. The experimental design levels and anaerobic digestion parameters are shown in Table 2. According to the experimental design, 17 runs were carried out. The anaerobic digesters were set up in triplicates for each treatment, and the findings were presented as means. Each factor was coded at five distinct levels and given the letters − α, − 1, 0, + 1, and + α as shown in Table 2. Biogas yield was used as the response of the experiment. The effectiveness of the second-order polynomial equation fit was expressed using the coefficient of determination (*R*^2^). Model terms were assessed using *P*-value [18, 20, 22, 23].

**Table 2:** Experimental levels of independent factors for the optimization of biogas production

Factor	Parameters	Levels				
		− α	− 1	0	1	+ α
*X*_1_	Substrate ratio (g) (WH: FW)	8:92	25:75	50:50	75:25	92:8
*X*_2_	Inoculum concentration (g)	1.6	5	10	15	18.4
*X*_3_	Dilution (mL)	81.6	85	90	95	98.4

## Results and discussion

### Statistical analysis and model fitting

The CCD of experimental variables in the actual and coded values and experimental results of the biogas yield is shown in Table 3. The analysis of variance (ANOVA) is shown in Table 4. All linear terms (*X*_1_, *X*_2_, *X*_3_) and quadratic terms (*X*_1_^2^, *X*_2_^2^, *X*_3_^2^) for all factors significantly affected the biogas yield because the *P*-value is less than 0.05 (*P* < 0.05) as shown in Table 4. The interaction between substrate ratio and IC (*X*_1_*X*_2_), and substrate ratio and dilution (*X*_1_*X*_3_) also significantly affected the biogas yield, while only the interaction between IC and dilution (*X*_2_*X*_3_) insignificantly affected the biogas yield because the *P*-value is greater than 0.05 (*P* > 0.05) as shown in Table 4. The model equation (Eq 1) was obtained based on multiple regression analysis for biogas production, and yielded the following quadratic model:

1 $$Y=-{6129.30019}+{32.47516{X_1}}+{165.91014{X_2}}+{109.98071{X_3}}-{0.485000{X_1}{X_2}}-{0.191000{X_1}{X_3}}-{0.157252{X_1^2}}-{6.14100{X_2^2}}-{0.523040{X_3^2}}$$

where *Y*: estimated Biogas Yield (response), *X*_1_: Substrate (WH: FW) ratio, *X*_2_:IC, and *X*_3_:Dilution. The model was significant (*P* < 0.05), this means that the quadratic model equation significantly affected the biogas yield. The lack of fit was insignificant (*P* > 0.05), this implies that the quadratic model significantly predicted the biogas yield. The coefficient of determination (*R*^2^) of 99.9% confirmed the good fit of the model with experimental variables.

**Table 3:** Experimental and predicted data

Std	Run	Coded values			Actual values			Actual values	Predicted values
		*X*_1_	*X*_2_	*X*_3_	*X*_1_	*X*_2_	*X*_3_		
13	1	0	0	α−	50	10	81.6	573	563.11
12	2	0	α+	0	50	18.41	90	325.5	322.47
14	3	0	0	2	50	10	98.41	662	657.86
11	4	0	− 2	0	50	1.591	90	115	104
2	5	1	− 1	− 1	75	5	85	231	240.09
7	6	− 1	1	1	25	15	95	690	690.83
5	7	− 1	− 1	1	25	5	95	434.5	442.93
4	8	1	1	− 1	75	15	85	250.5	251.99
10	9	2	0	0	92.04	10	90	152	147.07
16	10	0	0	0	50	10	90	648	647.47
17	11	0	0	0	50	10	90	648.5	647.47
6	12	1	− 1	1	75	5	95	249.5	251.93
9	13	− 2	0	0	7.955	10	90	601	591.9
8	14	1	1	1	75	15	95	254	257.33
3	15	− 1	1	− 1	25	15	85	582.5	589.99
1	16	− 1	− 1	− 1	25	5	85	329	335.59
15	17	0	0	0	50	10	90	643.5	647.47

Where: *X*_1_, *X*_2_, and *X*_3_ are the coded values of substrate ratio, IC, and dilution, respectively

**Table 4:** ANOVA for response surface polynomial model

Source	Sum of squares	df	Mean square	*F*-value	*P*-value
Model	6.67E+05	9	74,136.94	807.7	< 0.0001 significant
*X*_1_-Substrate ratio	2.39E+05	1	2.39E+05	2602.33	< 0.0001
*X*_2_-IC	57,612.09	1	57,612.09	627.67	< 0.0001
*X*_3_-Dilution	10,835.46	1	10,835.46	118.05	< 0.0001
*X*_1_*X*_2_	29,403.13	1	29,403.13	320.34	< 0.0001
*X*_1_*X*_3_	4560.13	1	4560.13	49.68	0.0002
*X*_2_*X*_3_	21.13	1	21.13	0.2302	0.646
*X*_1_^2^	1.09E+05	1	1.09E+05	1186.38	< 0.0001
*X*_2_^2^	2.66E+05	1	2.66E+05	2894.89	< 0.0001
*X*_3_^2^	1927.55	1	1927.55	21	0.0025
Residual	642.51	7	91.79		
Lack of fit	627.34	5	125.47	16.55	0.058 not significant
Pure error	15.17	2	7.58		
Cor total	6.68E+05	16			

Where *R*^2^ = 0.999, Adjusted *R*^2^ = 0.9978, Predicted *R*^2^ = 0.9928, Adequate Precision = 79.8627, and C.V = 2.2

A strong model fit is indicated by an *R*^2^ value between 0.75 and 1.0 for a good statistical model. The quadratic equation could be used to obtain a precise estimate for biogas production because of the high value of *R*^2^. According to Chanathaworn [18], the adjusted *R*^2^ of 0.9978 indicated that the response surface model created for this biogas study prediction was completely appropriate. A value greater than 4 is desirable for the "Adequate precision," which measures the signal-to-noise ratio, the ratio of 79.8627 from this study indicated an adequate signal. The Predicted *R*^2^ value of 0.9928 showed a good agreement between the predicted and observed values. The low coefficient of variation (CV) of 2.20 showed the high reliability and precision of experimental outcomes. The trustworthiness of experimental results decreases with an increased coefficient of variance (CV) [20, 23].

**Fig. 4:**
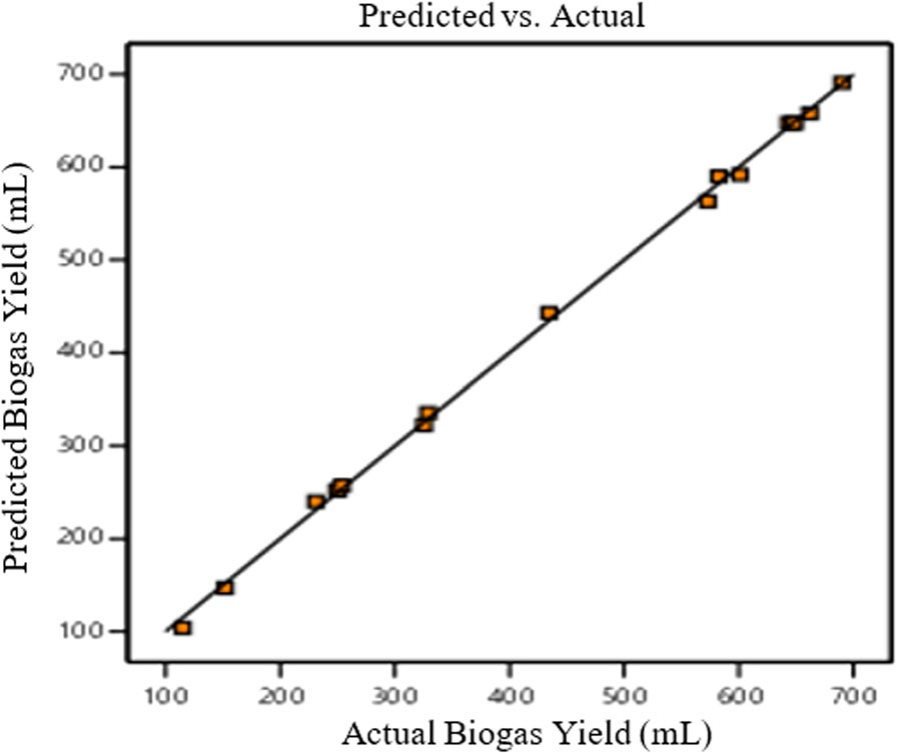
Plot of predicted response vs. actual value from response surface

A strong model fit is indicated by an *R*^2^ value between 0.75 and 1.0 for a good statistical model. The quadratic equation could be used to obtain a precise estimate for biogas production because of the high value of *R*^2^ [24]. According to Chanathaworn [19], the adjusted *R*^2^ of 0.9978 indicated that the response surface model created for this study’s biogas prediction was completely appropriate. A value greater than 4 is desirable for the "Adequate precision," which measures the signal-to-noise ratio, the ratio of 79.8627 from this study indicated an adequate signal. The Predicted *R*^2^ value of 0.9928 showed a good agreement between the predicted and observed values. The low coefficient of variation (CV) of 2.20 showed the high reliability and precision of experimental outcomes. The trustworthiness of experimental results decreases with an increased coefficient of variance (CV) [20, 21]. The experimental biogas production results were close to the predicted results as shown in Fig. 4.

### Analysis of response surfaces

**Fig. 5:**
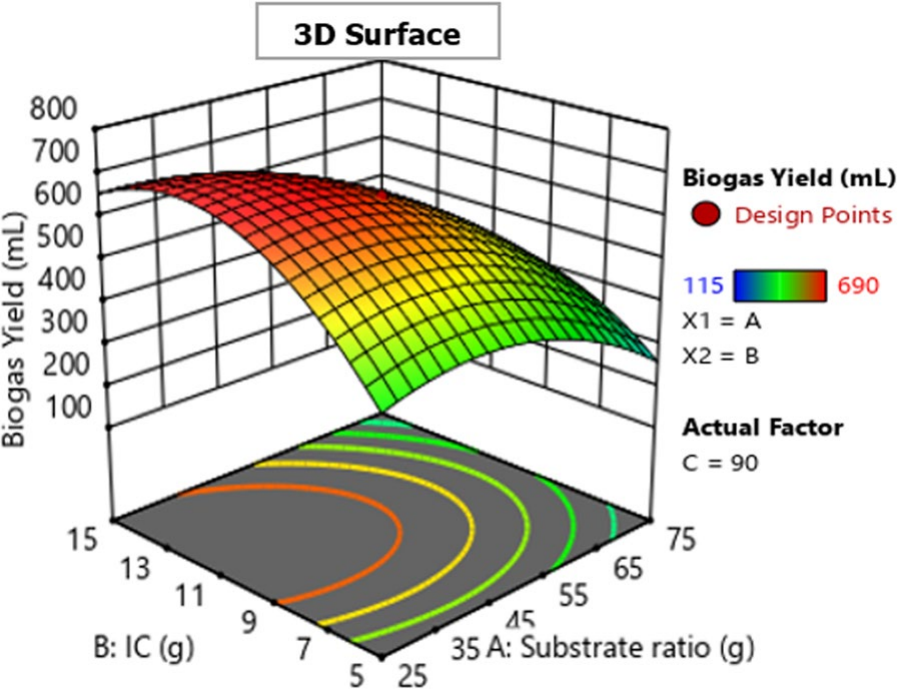
Effect of substrate ratio and IC on biogas production for response surface

**Fig. 6:**
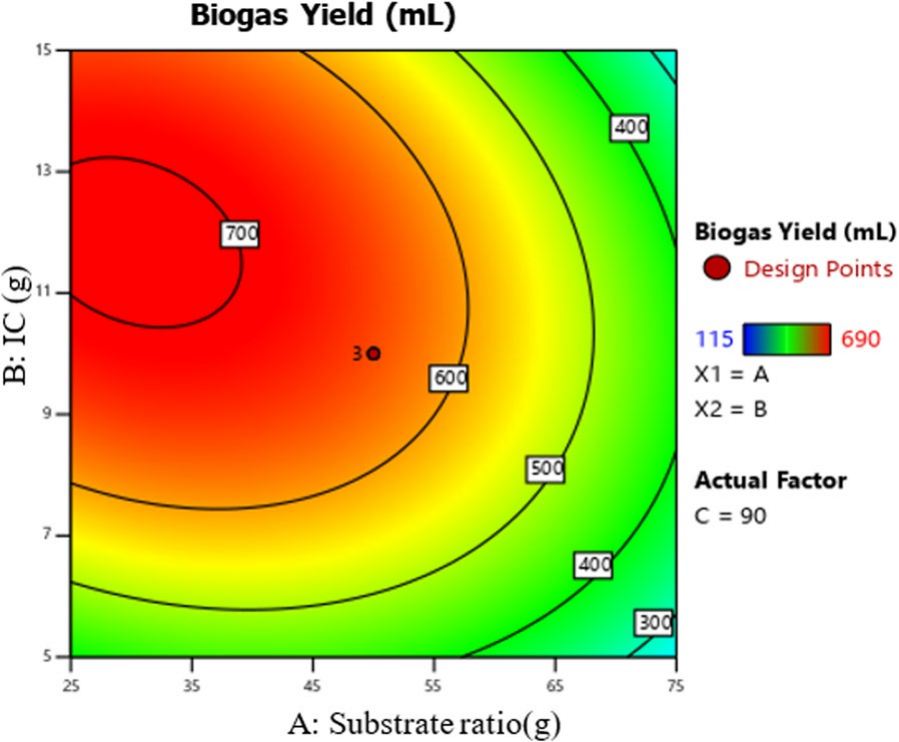
Effect of substrate ratio and IC on biogas production for contour plot

**Fig. 7:**
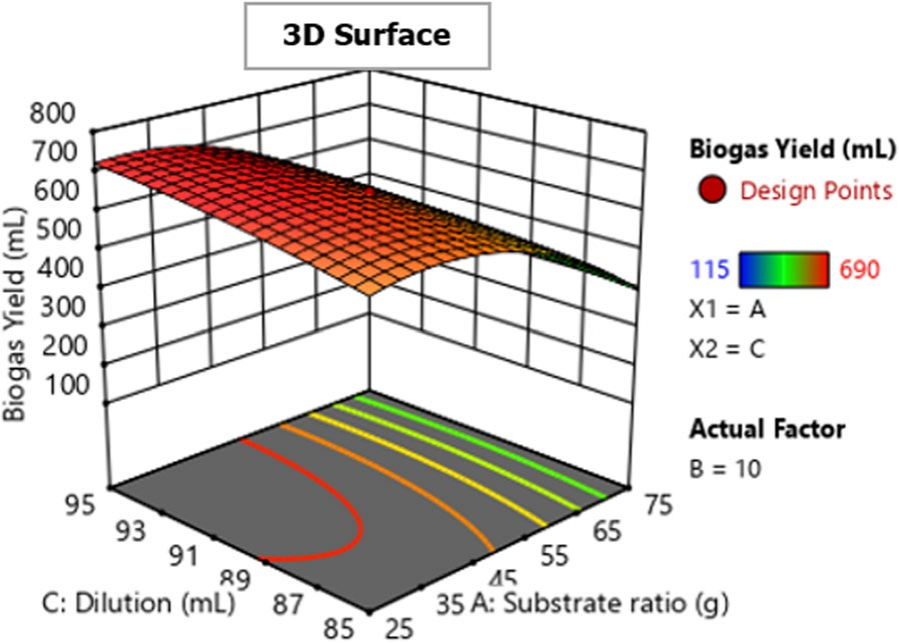
Effect of substrate ratio and dilution on biogas production for response surface

**Fig. 8:**
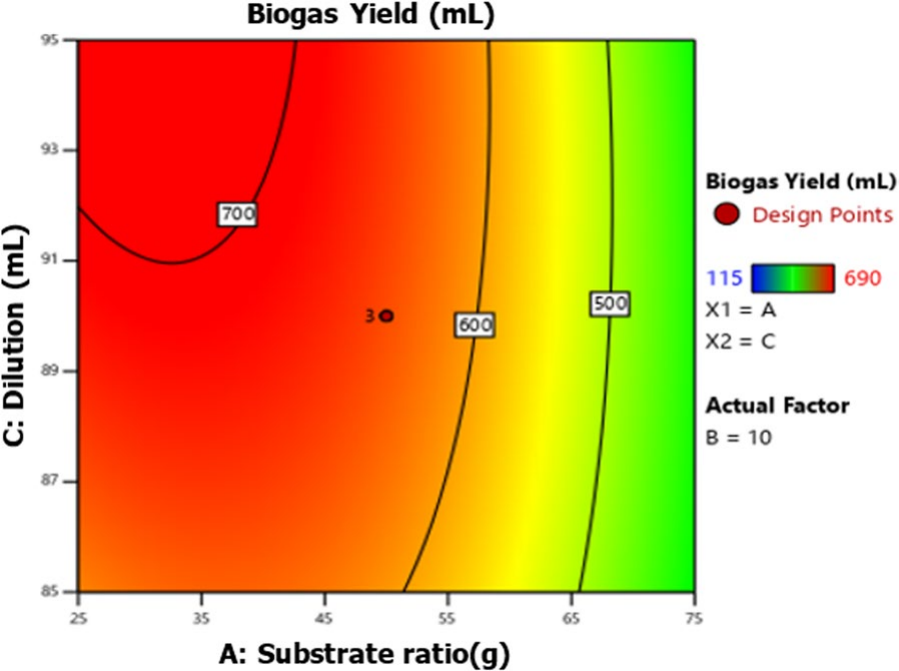
Effect of substrate ratio and dilution on biogas production for contour plot

**Fig. 9:**
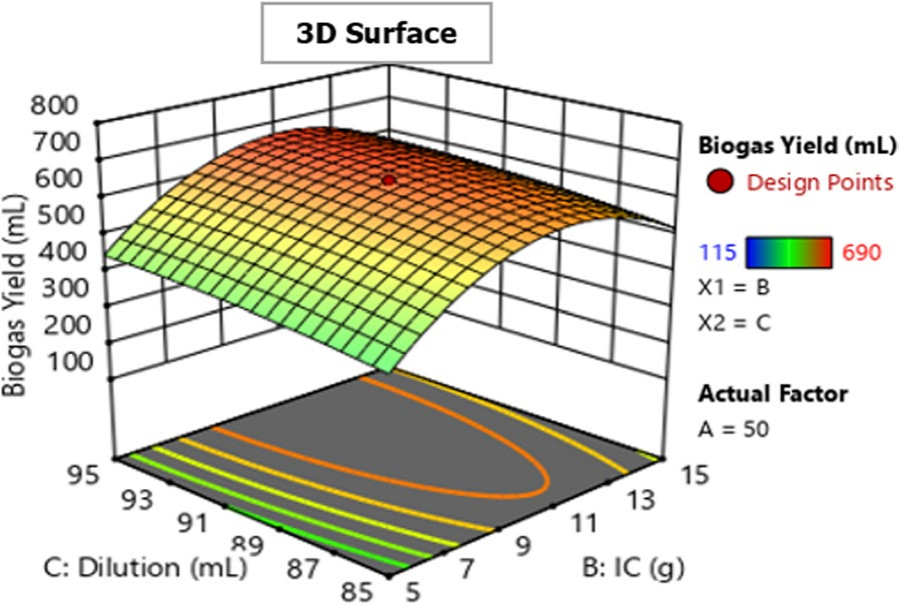
Effect of IC and dilution on the production of biogas for response surface

**Fig. 10:**
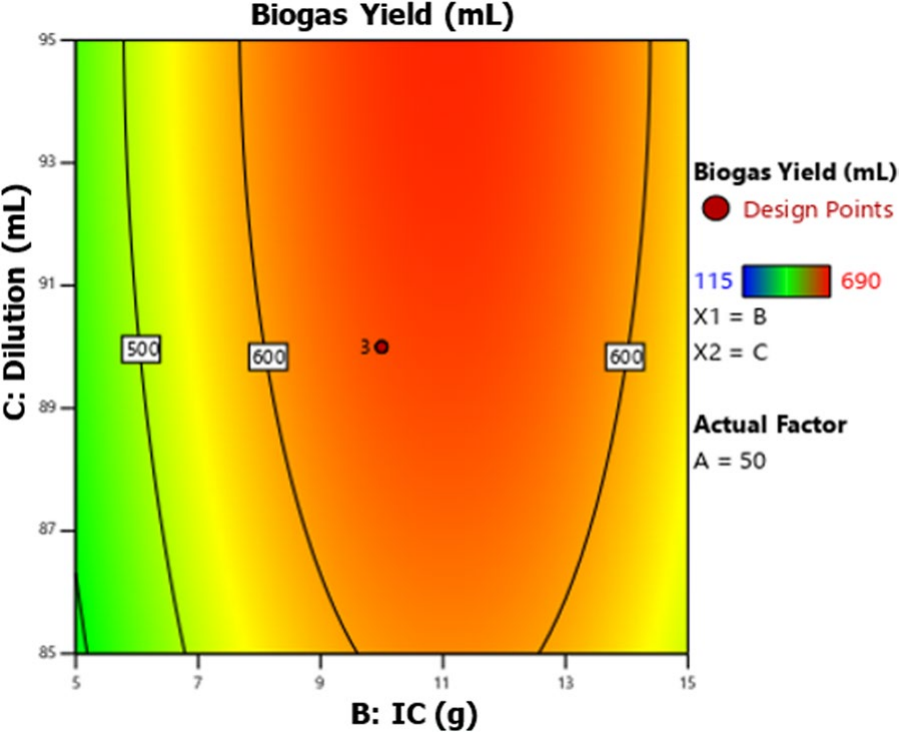
Effect of IC and dilution on the production of biogas for contour plot

The 2D (two-dimensional) contour and 3D (three-dimensional) response surfaces plots for biogas production optimization were represented using the polynomial model Eq. 1 to show the interaction effect of biogas production variables on the biogas yield. Figures 5, 6, 7, 8, 9 and 10 all display the 3D and 2D surface response plots.

#### Effect of substrate ratio and IC on the production of biogas

Effect of substrate ratio and inoculum concentration on biogas produced were determined as shown in Figs. 5 and 6. Biogas yield increased to its maximum when the IC increased. However, biogas production decreased when the substrate ratio increased as shown in Figs. 5 and 6. ANOVA showed that the interaction effect between substrate ratio and IC on biogas production was significant (*P* > 0.05) as shown in Table 4. Biogas production increased when the substrate ratio (WH:FW) was 25:75 g, however, when the substrate ratio (WH:FW) exceeds 25:75 g, respectively the biogas production decreased rapidly as shown in Fig. 4 and also when the substrate ratio (WH:FW) was less than 25:75 g, respectively a slight or very little inhibition was observed on the response surface plot. This might be explained by the presence of an insufficient amount of methanogens. This inhibition was due to the formation of intermediate products which are inappropriate for conversion by methanogenic bacteria to biogas and when there is overloading, the production of organic acids increases quickly, then inhibition of methanogens activity [11]. Similar findings were reported by Shen et al., Jnr et al., Labatut et al, Rabii et al., [25–28], overloading caused the microbial activity to be inhibited, which decreased the rate of biogas generation. The optimum biogas production of 690 mL with the methane content of 68.15% was obtained at 25:75 g of substrate ratio (WH:FW) when IC and dilution were 15 g and 95 mL, respectively as shown in Figs. 5 and 6.

#### Effect of substrate ratio and dilution on biogas production

The relationship between substrate ratio and dilution in biogas production is shown in Figs. 7 and 8.The results revealed that the interactive effect of substrate ratio and dilution on biogas production is significant (*P* < 0.05) as shown in Table 4. The biogas production increased as dilution increased and decreased when the substrate ratio increased. This is because a higher ratio of the substrate may cause an acidic environment in the AD system, which leads to methanogenesis inhibition, thus decreasing biogas production [25–28]. The optimum dilution for biogas production was 95 mL which obtained at substrate ratio (WH:FW) of 25:75 g and IC of 15 g.

#### Effect of IC and dilution on the production of biogas

The interaction effect of IC and dilution on biogas yield was insignificant (*P* > 0.05) as shown in Table 4. However, ANOVA indicated that the quadratic and linear terms of IC and dilution were significant (*P* < 0.05). Figures 9 and 10 show the relationship between IC and dilution in biogas generation. Biogas production was higher when IC was 11 g, however, when IC was less than 11 g the biogas production decreased rapidly, and also when IC exceeds 11 g a slight inhibition was observed as shown in Fig. 9. Biogas production was higher when IC was 11 g, however, when IC was less than 11 g the biogas production decreased rapidly, and also when IC exceeds 11 g a slight inhibition was observed as shown in Fig. 9. The biogas yield was very low when the IC was 1.5% and 18.4%. This might be explained by the presence of an insufficient amount of methanogens. Similar observations were reported by Dar and Phutela [14], at lower IC, there aren't enough bacteria present to start the methanogenesis. The results agree with Filer et al. and Girmaye et al. [22], the low inoculum concentration in the reactor could result in the microorganisms' low metabolic activity which leads to inhibition of the methanogenesis process resulting in low biogas yield. It was noted that the generation of biogas was slightly reduced as a result of the significant increase in IC (18.4%). This might have happened as a result of modifications to the substrates characteristics, which may have had an impact on the bioavailability during hydrolysis [11]. The addition of the required IC in the AD process is very important as it will enhance biogas yield and methane content, speed up the process, and improve the stability of anaerobic digestion [11, 26].

## Conclusion and future works

### Conclusion

The biogas yield was expressed as function of operating variables using a quadratic equation. The model was significant (*P* < 0.05). All factors had significant linear and quadratic effects on biogas while only the interaction effects of the two factors were significant. The coefficient of determination (*R*^2^) of 99.9% confirms the good fit of the model with experimental variables. Optimum values for RSM were within the range of experimental results. Biogas yield decreased as substrate ratio increased. According to the high value of *R*^2^, the model could be effectively utilized for the prediction of biogas generation from anaerobic co-digestion of FW and WH.

### Further research works

FW had a lower C/N ratio, further study needs to consider co-digestion with other higher C/N ratio substrates. Because the CO_2_ is hazardous to humans and corrodes motors and pipes, its removal is crucial. The biogas was not upgraded, research is still needed in purifying or upgrading the biogas for CO_2_ removal and improved methane content to be used directly for cooking or as fuel for vehicle.

## Data Availability

The data sets supporting the conclusions are included in this article. This paper also comprises all obligatory evidences. Upon demand, the corresponding author will deliver any supplementary data.
